# Clinical prediction and diagnosis of neurosyphilis in HIV-negative patients: a case-control study

**DOI:** 10.1186/s12879-019-4582-2

**Published:** 2019-12-02

**Authors:** Yong Lu, Wujian Ke, Ligang Yang, Zhenyu Wang, Ping Lv, Jing Gu, Chun Hao, Jinghua Li, Yumao Cai, Mei Gu, Hongfang Liu, Wenjing Chen, Xiaohui Zhang, Liuyuan Wang, Yahui Liu, Bin Yang, Huachun Zou, Heping Zheng

**Affiliations:** 1 School of Public Health, Sun Yat-sen University, Guangzhou, 510080 Guangdong China; 2 School of Public Health (Shenzhen), Sun Yat-sen University, Shenzhen, 518107 Guangdong China; 3 Dermatology Hospital, Southern Medical University, Guangzhou, 510091 Guangdong China; 4 Shenzhen Center for Chronic Disease Control, Shenzhen, Guangdong China; 5 Qingyuan Chronic Disease Prevention Hospital, Qingyuan, 511500 Guangdong China; 6 Kirby Institute, The University of New South Wales, Sydney, Australia

**Keywords:** Syphilis, Neurosyphilis, CSF, TPPA

## Abstract

**Background:**

Early diagnosis and treatment of neurosyphilis is of great significance for regression. There is no gold standard for the diagnosis of neurosyphilis. We did this study to explore the factors associated with the clinical diagnosis of neurosyphilis and assess their accuracy for the diagnosis of neurosyphilis.

**Methods:**

We retrospectively reviewed 100 cases of syphilis patients who underwent lumbar puncture at a major dermatology hospital in Guangzhou, China between April 2013 and November 2016. Fifty patients who were clinically diagnosed with neurosyphilis were selected as case group. Control group consisted of 50 general syphilis patients who were matched with age and gender. The records of patients were reviewed to collect data of socio-demographic information, clinical symptom, and laboratory indicators. Multivariable logistic regression was used to explore diagnostic indictors, and ROC analysis was used to assess diagnostic accuracy.

**Results:**

Neurological symptoms (odds ratio (OR) = 59.281, 95% CI:5.215–662.910, *P* = 0.001), cerebrospinal fluid (CSF) Treponema pallidum particle agglutination (TPPA) titer (OR = 1.004, 95% CI:1.002–1.006, *P* < 0.001), CSF protein (OR = 1.005, 95% CI:1.000–1.009, *P* = 0.041), and CSF white blood cell (WBC) (OR = 1.120, 95% CI:1.017–1.233, *P* = 0.021) were found to be statistically associated with neurosyphilis. In ROC analysis, CSF TPPA titer had a sensitivity of 90%, a specificity of 84%, and an area under curve (AUC) of 0.941.

**Conclusion:**

CSF TPPA can potentially be considered as an alternative test for diagnosis of neurosyphilis. Combining with neurological symptoms, CSF protein, CSF WBC, the diagnosis would have a higher sensitivity.

## Background

*Treponema pallidum (T. pallidum)* is the causative agent of syphilis, which can invade the central nervous system (CNS) at any stage after exposure [1, 2]. In about 14 to 20% of cases, *treponema pallidum* affects the central nervous system and can lead to asymptomatic meningitis, which can further progress to severe and irreversible symptomatic neurosyphilis if left untreated [3]. Therefore, early diagnosis and treatment of neurosyphilis is of great significance for regression [4]. Because of the complex stages of occurrence, changing clinical symptoms and variable laboratory indicators of neurosyphilis, early diagnosis of neurosyphilis is difficult [5–7].

There is no gold standard for the diagnosis of neurosyphilis. One commonly used diagnostic criteria developed by the Centers for Disease Control and Prevention (CDC) of the United States mentioned that neurosyphilis can be divided into two categories. One is “confirmed” neurosyphilis which can be diagnosed by the criterion that a reactive Venereal Disease Research Laboratory test (VDRL) in cerebrospinal fluid (CSF). The other one is “presumptive” neurosyphilis which can be diagnosed by the following criteria: (1) a nonreactive VDRL in CSF, (2) elevated CSF protein or leukocyte count, and (3) clinical symptoms or signs consistent with neurosyphilis without alternate known causes accounting for these [8]. According to the guideline of European CDC. CSF TT (Treponema pallidum haemagglutination assay (TPHA)/ Treponema pallidum particle agglutination (TPPA)) and intrathecal synthesis of immunoglobulins should be taken into consideration [9].

Although VDRL is considered as a definitive diagnosis test of neurosyphilis, there are some limitations. First, while VDRL has a high specificity, its sensitivity ranges from as low as 27 to 70% [10–12]. A nonreactive result of VDRL can not rule out the possibility of neurosyphilis. In these cases, CSF white blood cell (WBC), CSF protein, and the clinical symptoms should be taken into consideration. Second, VDRL test requires specialized glass plates and a light microscope. It may be hard to meet these requirements in resource-limited settings [13]. Third, the process of VDRL is time-consuming and complex [4]. Some previous studies suggested that clinical symptoms and some laboratory indicators maybe helpful to identify neurosyphilis patients. The objective of our study was to explore the factors associated with the clinical diagnosis of neurosyphilis and assess their accuracy for the diagnosis of neurosyphilis.

## Methods

### Study design and ethics statement

This study was conducted at a major dermatology hospital in Guangzhou, China. We retrieved data on syphilis patients who underwent the lumbar puncture between April 2013 and November 2016. In this study, we included 50 patients who were clinically diagnosed with neurosyphilis (NS), and then 50 general syphilis patients with the matched age ranges and gender ratio were randomly selected as control group. The exclusion criteria were as follows: Human Immunodeficiency Virus (HIV) infection, return visit, without lumbar puncture. The records of patients were reviewed to collect data such as age, gender, native place, occupation, marital status, HIV infection status, serum toludine red unheated serum test (TRUST) titer, serum TPPA titer, serum treponema pallidum immunoglobulin M (TP-IgM), CSF VDRL, CSF TRUST titer, CSF TPPA titer, CSF protein, CSF WBC, CSF glucose, CSF chlorides, and neurological symptoms (headache, visual symptoms, hypoacusis, seizures, motor function disorder, gait abnormalities, etc.). We only collected the results of laboratory tests within 90 days before or after the lumbar puncture. If a patient underwent multiple laboratory tests, we selected the results of the first test conducted before treatment into analysis. This study was approved by the Ethics Committee of the Dermatology Hospital of Southern Medical University. The ethics committee approval number is GDDHLS-20171004.

### Sample size calculation

Before we started our study, we have calculated the sample size. According to previous studies, we assumed that CSF WBC and CSF protein could be used as diagnostic indictors of neurosyphilis [5]. We selected the formula for calculating sample size for a case-control study with exposure variable as continuous variable. The formula is as follows: $n=\left(\frac{r+1}{r}\right)\frac{\sigma^2{\left({Z}_{\beta }+{Z}_{\alpha /2}\right)}^2}{(Difference)^2}$ [14]. In this formula, *r* is ratio of controls to cases, *σ* is the standard deviation of the variable which were compared, *difference* is the difference between the means of the case group and the control group, *α* is significance level, *β* is desired power. We used CSF WBC and CSF protein to calculate the sample size separately, and chose the larger one which was calculated with parameters of CSF protein as the sample size of our study. In the calculation process, *α* = 0.05, *Z*_*α/2*_ = 1.96, *β* = 0.8, *Z*_*β*_ = 0.84, *r* = 1, *Differenc*e = 150 mg/L, σ = 350 mg/L. The values of *Difference* and *σ* were derived from previous research [5]. The sample size was calculated as 86.

### Diagnostic criteria

The diagnosis of neurosyphilis were based on the guidelines of the Centers for Disease Control in Europe and America [8, 9]. The diagnosis criteria of neurosyphilis are as follows: 1.) a reactive VDRL in CSF or 2.) a negative VDRL in CSF with either elevated CSF protein (> 450 mg/L) or CFS WBC count (> 5 cells/μL) 3.) clinical symptoms or signs consistent with neurosyphilis without alternate known causes accounting for these. In the 50 neurosyphilis patients, 32 patients were diagnosed by criteria 1, and 18 patients were diagnosed by criteria 2.

### Statistical analysis

Reciprocal and logarithmic transformation were performed to titer data before data analysis.

Median and interquartile range (IQR) were used to describe continuous variables, while frequencies and percentages were used to describe categorical variables. The Mann-whtiney U test was used to compare continuous variables and the chi-square test was used to compare categorical variables. Logistic regression was used to calculate the odds ratio (OR) of laboratory indicators and clinical symptoms. We conducted a univariable analysis firstly. The variables which *P* value< 0.1 were retained. The multivariable models were created through stepwise elimination of variables from univariable analysis. In the multivariable analysis, odds ratios were adjusted for age and gender. Then the receiver operating characteristics (ROC) analysis was used to assess the indicators which were significant in logistic regression. Accuracy for the diagnosis of neurosyphilis. We used SPSS 20.0 and MedCalc 15.10 to perform statistical analyses. *P* values less than 0.05 were considered to be statistically significant.

## Results

### Characteristics of the study population

The characteristics of the 100 patients were shown in Table 1. The median age of study population was 47 (IQR, 40–52) years. There was no significant difference in age between the Non-NS group and the NS group (*p* = 0.953). Overall, 75% (75/100) of the study population were males, accounting for 68% in the Non-NS group and 82% the NS group (*p* = 0.106). In all patients, 84% were from Guangdong Province, with 82% in the Non-NS group and 86% in the NS group (*p* = 0.585). The Non-NS group had more patients who were single (18%) than the NS group (4%) (*p* = 0.025). Sixty two percent in the Non-NS group and 54% in the NS group (*p* = 0.418) had a permanent job. More patients (46%) in the NS group had neurological symptoms than patients (10.2%) in the Non-NS group (*p* < 0.001). Patients in the NS group had significantly higher serum TRUST titer (median, 1:16 versus 1:4, *p* < 0.001), Serum TPPA titer (median, > 1:1280 versus 1:1280, *p* = 0.006), Serum TP-IgM positive rate (positive rate, 53.7% versus 27.3%, *p* = 0.022), CSF TRUST titer (median, 1:2 versus Negative, *p* < 0.001), and CSF TPPA titer (median, 1:1280 versus Negative, *p* < 0.001) than patients in the Non-NS group. CSF protein and CSF WBC count were higher and CSF glucose and chlorides levels lower in the NS group than in the Non-NS group (*p* < 0.05 for all).

**Table 1:** Demographic and clinical characteristics of the study population

Characteristics	Total (*n* = 100) Median (IQR)/N (%)	Non-NS group (*n* = 50) Median (IQR)/N (%)	NS group (*n* = 50) Median (IQR)/N (%)	*p* value
Age, years	47(40–52)	47(40–52)	47(40–54)	0.953
Gender				0.106
Female	25 (25.0%)	16(32.0%)	9(18.0%)	
Male	75 (75.0%)	34(68.0%)	41(82.0%)	
Domicile place				0.585
Guangdong Province	84 (84.0%)	41(82.0%)	43(86.0%)	
Other provinces	16 (16.0%)	9(18.0%)	7(14.0%)	
Marital status				0.025
Single	11 (11.0%)	9(18.0%)	2(4.0%)	
Married	89 (89.0%)	41(82.0%)	48(96.0%)	
Occupation				0.418
Permanent job	58 (58.0%)	31(62.0%)	27(54.0%)	
Temporary job	42 (42.0%)	19(38.0%)	23(46.0%)	
Neurological symptoms	28 (28.3%)	5(10.2%)	23(46.0%)	< 0.001
Serum TRUST titer	1:8 (1:4–1:16)	1:4 (1:2–1:16)	1:16(1:8–1:32)	< 0.001
Serum TPPA titer	1:1280 (> 1:1280–1:1280)	1:1280 (1:1280–1:1280)	> 1:1280 (> 1:1280–1:1280)	0.006
Serum TP-IgM	31(41.9%)	9(27.3%)	22(53.7%)	0.022
CSF TRUST titer	Neg (Neg- Neg)	Neg (Neg- Neg)	1:2 (Neg-1:4)	< 0.001
CSF TPPA titer	1:640 (Neg-1:1280)	Neg (Neg-1:160)	1:1280 (1:1280–1:1280)	< 0.001
CSF protein, mg/L	365.0 (270.0–598.9)	377.6(237.0–421.2)	521.5 (300.5–796.0)	< 0.001
CSF WBC, cells/μL	1 (0–5)	0(0–5)	3(0–10)	0.005
CSF glucose, mmol/L	3.61 (3.30–4.00)	3.70 (3.35–4.00)	3.5(3.2–3.9)	0.036
CSF chlorides, mmol/L	120.0 (117.0–122.0)	121.0(117.8–124.5)	118.1(115.8–122.0)	0.009

*NS* Neurosyphilis, *TRUST* Toludine red unheated serum test, *TPPA* Treponema pallidum particle agglutination, *TP-IgM* Treponema pallidum IgM, *CSF* Cerebrospinal fluid, *Neg* Negative, *WBC* White blood cell. Median and interquartile range (IQR) were used to describe continuous variables, while frequencies and percentages were used to describe categorical variables

### Predictors of neurosyphilis

Univariable logistic regression indicated that the following variables were significantly associated with NS: Neurological symptoms, Serum TPPA titer, Serum TP-IgM, CSF TPPA titer, CSF protein, CSF WBC, CSF chlorides (Table 2).

**Table 2:** Predictors of neurosyphilis according to univariable logistic regression

Variable	Coefficient	OR	95% CI	*p* value
Neurological symptoms	2.014	7.496	2.547–22.059	< 0.001
Serum TRUST titer	0.014	1.014	0.992–1.036	0.211
Serum TPPA titer	0.003	1.003	1.000–1.006	0.030
Serum TP-IgM	1.127	3.088	1.157–8.241	0.024
CSF TPPA titer	0.004	1.004	1.003–1.005	< 0.001
CSF protein	0.004	1.004	1.002–1.006	0.001
CSF WBC	0.071	1.074	1.002–1.152	0.045
CSF glucose	−0.072	0.931	0.620–1.398	0.729
CSF chlorides	−0.111	0.895	0.820–0.976	0.012

*OR* Odds ratio, *CI* Confidence interval, *TRUST* Serum toludine red unheated serum test, *TPPA* Treponema pallidum particle agglutination, *TP-IgM* Treponema pallidum IgM, *CSF* Cerebrospinal fluid, *WBC* White blood cell

Then, the results of multivariable logistic regression showed only the following four variables were included in the model: neurological symptoms (OR = 59.281, 95% Confidence interval (CI):5.215–662.910, *p* = 0.001), CSF TPPA titer (OR = 1.004, 95% CI:1.002–1.006, *p* < 0.001), CSF protein (OR = 1.005, 95% CI:1.000–1.009, *p* = 0.041), and CSF WBC (OR = 1.120, 95% CI:1.017–1.233, *p* = 0.021) (Table 3). Patients with neurological symptoms were 59.281-fold more likely to be diagnosed as neurosyphilis. When CSF TPPA titer doubled, patients were 1.004-fold more likely to be diagnosed as neurosyphilis. When CSF protein increased 1 mg/L, patients were 1.005-fold more likely to be diagnosed as neurosyphilis. When CSF WBC increased 1 cells/μL, patients were 1.120-fold more likely to be diagnosed as neurosyphilis.

**Table 3:** Predictors of neurosyphilis according to multivariable logistic regression

Variable	Coefficient	Adjusted OR^a^	95% CI	*p* value
Neurological symptoms	4.082	59.281	5.215–662.910	0.001
CSF TPPA titer	0.004	1.004	1.002–1.006	< 0.001
CSF protein	0.005	1.005	1.000–1.009	0.041
CSF WBC	0.113	1.120	1.017–1.233	0.021

*OR* Odds ratio, *CI* Confidence interval, *CSF* Cerebrospinal fluid, *TPPA* Treponema pallidum particle agglutination, *WBC* White blood cell

^a^Odds ratios were adjusted for age and gender

### Sensitivity and specificity analyses of neurological symptoms, CSF TPPA titer, CSF protein, and CSF WBC

**Fig. 1:**
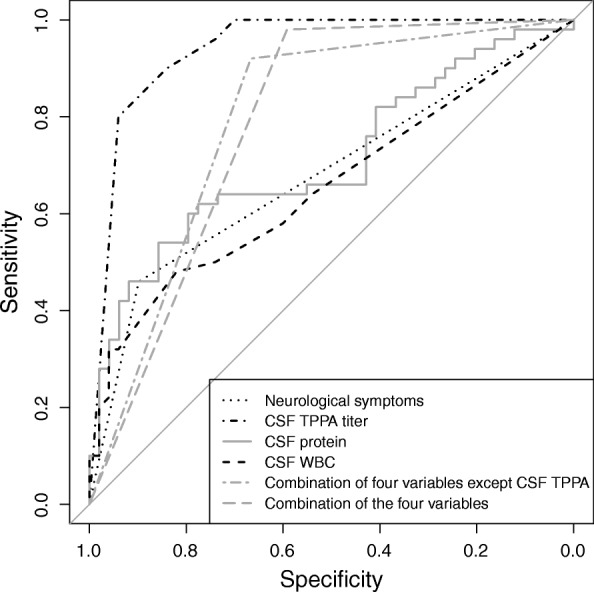
Receiver operating characteristic curve for syphilitic indicators

We conducted ROC analyses to assess the diagnosis accuracy of neurological symptoms, CSF TPPA titer, CSF protein, and CSF WBC. For different cutoff points, the ROC analyses showed different sensitivity, specificity and area under curve (AUC). The AUC was the best, when CSF TPPA titer was at 1:160, CSF protein was at 497 mg/L, and CSF WBC was at 3 cells/μL, respectively. The AUC of neurological symptoms was 0.679 (Fig. 1), and 95% CI was 0.578–0.769; sensitivity was 46.00%, while specificity was 89.80%. The AUC of CSF TPPA titer was 0.941, and 95% CI was 0.876–0.978; sensitivity was 90.00%, while specificity was 84.00%. The AUC of CSF protein was 0.710, and 95% CI was 0.610–0.797; sensitivity was 54.00%, while specificity was 85.71%. The AUC of CSF WBC was 0.655, and 95% CI was 0.553–0.747; sensitivity was 48.00%, while specificity was 82.00% (Table 4). Compared with the other three variables, CSF TPPA titer had the highest AUC (*P* < 0.001). However, there were no significant differences in AUC among neurological symptoms, CSF protein, and CSF WBC (*P* > 0.05). When we combined neurological symptoms, CSF protein, and CSF WBC (i.e. when the patient had neurological symptoms or any of the indicators (i.e. CSF protein and CSF WBC) exceeded the cutoff values, the patient was considered to be neurosyphilis), the sensitivity and specificity were 92.00 and 33.30%, respectively. When we combined neurological symptoms, CSF protein, CSF WBC, and CSF TPPA using the same method mentioned above, the sensitivity and specificity were 98.00 and 40.80%, respectively. We transformed the CSF TPPA titer, CSF protein, and CSF WBC into dichotomous variables by cutoff values and then performed logistic regression analysis. The results of multivariable logistic regression also showed neurological symptoms (OR = 46.920, 95% CI:2.945–747.637, *p* = 0.006), CSF TPPA titer (OR = 76.000, 95% CI:16.030–360.323, *p* < 0.001), CSF protein (OR = 30.569, 95% CI:2.121–440.487, *p* = 0.012), and CSF WBC (OR = 5.540, 95% CI:1.096–27.995, *p* = 0.038) were included in the model.

**Table 4:** The evaluation of diagnostic tests of syphilitic indicators for neurosyphilis

Variable	Sensitivity (%)	Specificity (%)	AUC & 95% CI	*p* value
Neurological symptoms	46.00	89.80	0.679 (0.578–0.769)	< 0.001
CSF TPPA titer (cutoff > 1:160)	90.00	84.00	0.941 (0.876–0.978)	< 0.001
CSF protein (cutoff > 497 mg/L)	54.00	85.71	0.710 (0.610–0.797)	< 0.001
CSF WBC (cutoff > 3 cells/μL)	48.00	82.00	0.655 (0.553–0.747)	0.003
A combination of the above tests except CSF TPPA	92.00	33.30	0.793(0.700–0.887)	< 0.001
A combination of the above tests	98.00	40.80	0.786 (0.692–0.880)	< 0.001

*AUC* Area under curve, *CSF* Cerebrospinal fluid, *TPPA* Treponema pallidum particle agglutination, *WBC* White blood cell

## Discussion

Neurosyphilis is still difficult to diagnose because there is no definitive test for diagnosis. Nowadays, CSF-VDRL is considered as widely used diagnostic criteria for neurosyphilis. However, VDRL still has some shortcomings, such as high specificity and low sensitivity [10–12], special equipment requirements [13], reagents need to be used within 2 hours [4]. Some of the major clinical guidelines suggest that we should take into account the results of some laboratory tests when we diagnose neurosyphilis, e.g. CSF WBC and CSF protein [15, 16]. Our result showed that neurological symptoms, Serum TPPA titer, Serum TP-IgM, CSF TPPA titer, CSF protein, CSF WBC, and CSF chlorides were predictors of neurosyphilis. The results of the multivariable logistic regression revealed that neurological symptoms, CSF TPPA titer, CSF protein, CSF WBC were independent predictors of neurosyphilis.

In our study, CSF TPPA titer had a sensitivity of 90%, a specificity of 84%, and an AUC of 0.941. The diagnostic accuracy was much higher than other three predictors. The results of our study were similar with some previous studies [4, 17]. Castro R et al. mentioned that the sensitivity of TPPA to diagnose neurosyphilis is 100%. In their study, 198 CSF samples from syphilis patients were studied. Among them, 133 were infected with HIV and 16 were neurosyphilis. The results of CSF TPPA were reactive in the 16 cases of neurosyphilis [17]. Another study conducted in China also showed the sensitivity of CSF TPPA was 100%. That study recruited 1132 syphilis patients. The results of CSF TPPA in 210 neurosyphilis patients were reactive. The above two studies suggested that reactive CSF TPPA without titer requirements could be considered as a diagnostic indicator of neurosyphilis [4, 17]. However, these studies did not show the specificity of CSF TPPA. In consideration of the high sensitivity, specificity and AUC of TPPA, we suggested that CSF TPPA can be used for the diagnosis of neurosyphilis.

Sensitivity of neurological symptoms, CSF protein, CSF WBC changed from 46 to 54%. These predictors were insensitive and nonspecific. A literature review indicated that part of early neurosyphilis patients presented neurological symptoms of typical aseptic meningitis, including headache, stiff neck, nausea, vomiting. The most common symptoms included papilledema, convulsions, confusion, and focal and cranial nerve abnormalities. In the advanced stage of neurosyphilis, the neurological symptoms of patients were usually dementia and tabes dorsalis. Meanwhile, there were still some asymptomatic neurosyphilis patients. So the indicator of neurological symptoms showed a high specificity and low sensitivity [18].

When we took neurological symptoms, CSF protein, CSF WBC into account at the same time, the sensitivity rose to 92.00%, and when we combined neurological symptoms, CSF protein, CSF WBC, and CSF TPPA, the sensitivity rose to 98.00%. There were some things to be noticed when we used these predictors to diagnose neurosyphilis. Previous studies mentioned that elevated CSF protein and WBC can occur in HIV-infected patients without neurosyphilis [3, 19, 20]. Thus, using higher cutoff values of CSF WBC and protein for diagnosis of neurosyphilis in HIV positive patients can improve specificity. In HIV negative patients, a cutoff value of > 5 cells/μL is usually used as a standard threshold of CSF WBC to diagnose neurosyphilis [3, 8]. However, previous studies conducted in China indicated that 10 cells/μL should be considered as threshold of CSF WBC [5]. Chinese CDC Guidelines also suggested ≥10 cells/μL as threshold of diagnosis of neurosyphilis [21]. And the results of our study showed the cutoff of CSF WBC should be>3cells/μL. There was no standard threshold of CSF protein used to diagnose neurosyphilis because of the different laboratory conditions [3]. The results of our study showed the cutoff of CSF protein should be>497 mg/L which was close to the threshold proposed by the U.S. CDC [8]. Compared with previous studies, the results of our study confirmed that neurological symptoms, CSF protein, CSF WBC, and CSF TPPA can be used alone or in combination as indicator for the diagnosis of neurosyphilis. More and large population studies should be conducted to confirm the standard threshold of CSF WBC, CSF protein and CSF TPPA.

Our study was limited by the design of retrospective study. The data for this study were collected from patients who underwent lumbar puncture examination, which may lead to potential bias in patient selection. Potential selection bias may exist since patients of control group were included base on matched age and gender. There was a possibility that some cases were misclassified because of the lack of gold standard for the diagnosis of neurosyphilis.

## Conclusions

CSF-VDRL is highly specific but insensitive as a widely used diagnostic method. When CSF-VDRL is nonreactive, some other indicators should be considered. In our study, neurological symptoms, CSF TPPA titer, CSF protein, CSF WBC were identified as independent predictors of neurosyphilis. Especially, CSF TPPA had high sensitivity, specificity and AUC. Therefore, we suggested that CSF TPPA should be considered as an alternative test for the diagnosis of neurosyphilis. Combining with neurological symptoms, CSF protein, CSF WBC, the diagnosis would have a higher sensitivity.

## Data Availability

The datasets used and/or analyzed during the current study are available from the corresponding author on reasonable request.

## Abbreviations

AUC: Area under curve
CDC: Centers for Disease Control and Prevention
CNS: Central nervous system
CSF: Cerebrospinal fluid
HIV: Human Immunodeficiency Virus
IQR: Interquartile range
NS: Neurosyphilis
OR: Odds ratio
ROC: Receiver operating characteristics
*T. pallidum*: Treponema pallidum
TPHA: Treponema pallidum haemagglutination assay
TP-IgM: Treponema pallidum immunoglobulin M
TPPA: Treponema pallidum particle agglutination
TRUST: Toludine red unheated serum test
VDRL: Venereal Disease Research Laboratory test
WBC: White blood cell
